# *Teucrium polium* Extract Enhances the Anti-Angiogenesis Effect of Tranilast on Human Umbilical Vein Endothelial Cells

**DOI:** 10.15171/apb.2018.016

**Published:** 2018-03-18

**Authors:** Fatemeh Sheikhbahaei, Mozafar Khazaei, Seyed Noureddin Nematollahi-Mahani

**Affiliations:** ^1^Department of Anatomy, Afzalipour School of Medicine, Kerman University of Medical Sciences, Kerman, Iran.; ^2^Fertility and Infertility Research Center, Kermanshah University of Medical Sciences, Kermanshah, Iran.; ^3^Physiology Research Center, Institute of Neuropharmacology, Kerman University of Medical Sciences, Kerman, Iran.

**Keywords:** Angiogenesis, Apoptosis, Teucrium polium, Tranilast, Human Umbilical Vein Endothelial Cells (HUVECs)

## Abstract

***Purpose:*** Angiogenesis plays an important role in numerous pathophysiological events like cancer. As a result of this, tranilast as an anti-fibrotic drug induces the promising antitumor activities through the inhibition of angiogenesis. Further, Teucrium polium (TP) is a herbal medicine (family Lamaceae) with antitumor properties. This study was conducted to investigate the combination effects of tranilast and T. polium on human umbilical vein endothelial cells (HUVECs) viability and apoptotic genes expression.

***Methods:*** The HUVECs line was treated using different doses of tranilast and T. polium alone or their combination. The cell cytotoxicity was evaluated using MTT and LDH assays; apoptosis was examined using acridine orange/ethidium bromide staining, nitric oxide (NO) production was evaluated using Griess reaction and the expression of BAX and BCL-2 genes were detected using real-time RT-PCR. One-way analysis of variance (ANOVA) test was used to compare the data in different groups.

***Results:*** The survival rate of HUVECs was significantly reduced (p<0.05) in a dose dependent manner by tranilast and T. polium. However, T. polium and tranilast combination significantly (p<0.001) reduced cell viability and increased apoptotic cells as compared to each drug alone. Also, HUVECs treated with Tranilast / T. polium combination showed a reduced level of NO as regards to cells exposed only to Tranilast or T. polium (p<0.05). Furthermore, a significant increase in BAX and a decrease in BCL-2 mRNA expression were observed in combination group (p<0.001).

***Conclusion:*** T. polium synergistically increased the antiangiogenic effect of tranilast on in vitro angiogenic model of HUVECs.

## Introduction

Angiogenesis targeting has recently been suggested as a promising approach for anticancer therapies.^1^ Branching of the vascular network and formation of new blood vessels from pre-existing vasculature identified as angiogenesis is considered as a key biological process, which involved physiologic conditions such as embryonic development, endometrial repair, ovulation, and pathologic changes including solid cancers growth, diabetic retinopathy and rheumatoid arthritis.^2^ It has been suggested that angiogenesis requires the balance between the activation of stimulating and inhibitory molecules that initiate or stop this phenomenon. Owing to this, factors such as hypoxia, decreased pH, increased lactic acid, mutations in oncogene and tumor suppressor genes seem to disturb the balance in an attempt to alter the process of angiogenesis.^3^ It is evident that in the absence of vascular support, apoptosis and/or necrosis may be activated in tumoral cells. When it is observed that tumor mass is required to achieve more nutrients and oxygen; angiogenesis signaling is stimulated to compensate for deprivation. Hence, inhibition of angiogenesis may be considered as an important way to combat cancer progression.^4^

Tranilast (N-[3,4-dimethoxycinnamonyl]-anthranilic acid) was first introduced by Koda *et al.* to suppress progression of homologous passive coetaneous anaphylaxis.^5^ It was later used for the treatment of hypertrophic scars and keloids. Some researchers found that tranilast is able to inhibit the proliferation of fibroblasts *in vitro* and selectively suppress collagen deposition *in vivo.*^6^ Also, it was developed as an anti-fibrotic and anti-allergic drug in Japan.^7^ Aside these, tranilast was found to decrease the proliferation of fibroblasts from normal tissues and suppress the release of *TGF-β* from fibroblasts.^8^

In addition , its antitumor activity has been reported in some cell lines such as PC-3, LNcap-SF and LNcap (prostate cancer cell lines),^9^ LN-18 and T98G (human malignant glioma cells).^10^ In addition, numerous studies have shown the antigrowth effect of tranilast on breast cancer cell lines like 4T, LA7, MDA-MB-231, MCF-7 and BT-474.^11,12^ It has also been shown to block cell cycle progression, inhibit migration and promote the apoptosis of MDA-MB-231 and BT-474 cells.^13^ On the other hand‏, it has been shown that this drug possesses antiangiogenic effect on human dermal microvascular endothelial cells by inhibition of the migration, proliferation ,tube formation, and expression of *VEGF* both *in vitro* and *in vivo*.^14^

Combination therapies with herbal extract have been proposed to reduce the resistance of several cancerous cells to chemical agents.^15^ *Teucrium polium (TP) is* a member of the *Lamaceae* family and has been used for over 2000 years in folk medicine because of its pharmacological properties.^16^ This herb consists of more than 300 species and is found in Europe and mainly in the Mediterranean area. Plants of this genus have been used for medicine since ancient Greek times, when they were used for coughs and asthma. *T. polium* has been shown to possess antioxidant, anti-inflammatory, antibacterial, antifungal, antiseptic, antipyretic and hypolipidemic properties.^17^

The anticancer properties of *T. polium* have been shown in some studies on a huge number of cancer cell lines, such as MCF-7 and MDA-MB-231 breast adenocarsinoma,^11,12^ bladder carcinoma, epidermoid carcinoma (A431), Skmel-3 Melanoma cell line, Saos-2 osteoblastoma, SW480 colon carcinoma,^18^ K562 chronic myelogenous leukemia,^19^ REYF-1 glioblastoma multiforme,^20^ BT20 human breast ductal carcinoma, PC12 mouse pheochromocytoma and A549 human lung adenocarsinoma cell lines.^21^ In addition to the antitumor properties of the crude extract of *T. polium*, different fractions of *T. polium* have also shown promising antitumor activity , namely, the petroleum ether fraction as the most potent fraction that was examined on U87 cells.^22^

A major part of the findings on endothelial cell functions is from *in vitro* experiments with HUVECs line. HUVECs are among the most widely used cells of *in vitro* angiogenesis models. These cells provide a classic model system to study numerous aspects of endothelial function and disease such as normal, abnormal and tumor-associated angiogenesis, oxidative stress, hypoxia and inflammation related pathways in endothelia under normal and pathological conditions.^23^ Typical characteristics of *in vitro* angiogenesis assays are proliferation, migration, and the formation of tube-like structures by endothelial cells.^24^

Nitric oxide (NO) is an important signaling factor in many pathological and physiological processes.^25^ It is secreted by the endothelium of blood vessels and plays an important role in the angiogenic cascade through the modulation of the activity of angiogenic factors released by tumor cells such as *VEGF*.^26^ It has been shown that (NO) is present at higher levels in solid tumors, when compared with normal tissues.^27^

Inhibition of angiogenesis as a new therapeutic option can be considered by following the pivotal role of angiogenesis in tumor growth. It has shown that highly vascular tumors have higher potential of metastasis and poorer prognoses. Since angiogenesis inhibition is an effective antigrowth strategy for treatment of tumor cells, for a causal role of medicinal herbs and the lack of scientific documented evidence regarding antiangiogenic of *T. polium*, attempt has been made to survey the effect of *T. polium* alone and in combination with tranilast on angiogenesis and proliferation of HUVECs. Altogether, the aim of the present study was to investigate possible synergistic antiangiogenic effect of *T. polium in* with tranilast, in with the expectation of creating a more effective anti-tumor treatment strategy.

## Materials and Methods

Tranilast was purchased from Sigma-Aldrich, (Mo, USA) and dissolved in dimethyl sulfoxide (DMSO) such thatthe final DMSO concentration in experimental wells did not exceed 0.5% (v/v). Dulbecco’s modified Eagle’s medium and Ham's F12 (DMEM/F12), fetal bovine serum (FBS), trypsin, and acridine orange (AO) were purchased from Sigma-Aldrich Chemical Co (St. Louis, MO, USA). Gene Matrix Universal RNA Purification Kit was purchased from EURx Ltd. Gdansk (Poland ul). PrimeScriptTM 1st strand cDNA Synthesis Kit and SYBR Premix Ex Taq technology were purchased from Takara (Bio Inc. Japan).

### Plant 

The areal parts of *T. polium* were collected from Khabr (Kerman, Iran) and identified at the Department of Pharmacognosy, Kerman University of Medical Sciences. A specimen was deposited at the herbarium of the Kerman School of Pharmacy (Voucher number: 28125).

The aerial parts of* T. polium* (200 g) were dried and made into powder. Ethanol extract was provided using maceration method in 80% ethanol for 24 h, followed by evaporation by rotatory evaporator at 50° C. The crude extract was fractionated using petroleum ether by solvent-solvent extraction method. The petroleum ether fraction was concentrated by a rotary evaporator and lyophilized in a freeze dryer.‏ The extract was dissolved in 10µl of DMSO, so that the final concentration of DMSO was adjusted to less than 0.05% in the culture medium.^22^

### Cell culture and treatment

(HUVECs) were obtained from the National Cell Bank (Pasteur Institute, Tehran, Iran). The cells were grown in a complete medium consisting of DMEM/F12 medium supplemented with 10% FBS in a humidified atmosphere of 5% CO_2_ at 37ºC.‏ The cells were treated with 75, 150, 300, 600, and 1200 µM tranilast‏ (base on pilot study) and 25, 50, 100, 200,and 400 µg/ml PE fraction of *T. polium* (base on pilot study*)* for 24, 48 and 72 h. Each treatment was replicated in at least three independent experiments and each was carried out in triplicates.

### MTT assay 

HUVECs (15× 10^3^ per well) were plated in 96 well culture plate‏.The wells were treated with different concentrations of tranilast and *T. polium* in culture medium, and incubated for 24, 48 and 72 h.. The viability of the cells was recorded at an absorbance of 570 nm and the reference wavelength of 630 nm using an ELISA reader. The percentage of cell viability= (Absorbance of treated cell / Absorbance of control cells) × 100, was calculated.^28^

### Median effect analysis 

Multiple drug effect analysis of Chou and Talaly, based on the median-effect principle, was used to calculate the combined drug effect. This method evaluates the nature of tranilast and *T. polium* interaction (synergistic, additive, or antagonistic). The combination of tranilast and *T. polium* in fixed concentration ratio based on their corresponding IC50 values (302.58 µM for tranilast and 98.7 µg/ml for *T. polium*) in two-fold serial dilutions above and below the IC50 value of agents was done. Combination index (CI) and dose reduction index (DRI) values were calculated using CompuSyn software (ComboSyn, Inc., Paramus, NJ, USA).

The CI values were interpreted as additive (CI = 1), synergistic (CI < 1) and antagonistic (CI > 1). DRI value is the degree to which the concentration of a mean compound can be reduced when used in combination with another compound to maintain an equivalent effect and “Fa” is the fraction of cell death ranging from 0 (no cell killing) to 1 (100% of cell killing). Classical isobolograms were also constructed by plotting drugs concentrations (alone and in combination) that inhibit 50, 75,and 90% viability. In this assay, CI>1 is the antagonist effect, CI = 1 is the additive effect, and CI < 1 is the synergistic effect.^28^

### LDH assay 

The cells (7×10^4^) were cultured in 4 well plates and treated with 1.5 ml medium containing tranilast and *T. polium*. Following 72 h incubation, the supernatant was collected and centrifuged at 250 g for 10 min and lactate dehydrogenase activity was measured following the manufacturer's instructions.^29^

### Acridine orange / ethidium bromide (AO/EB) staining 

HUVECs were cultured in 24 well plates and incubated for 24 h. The cells were then treated with 300µM tranilast and 100 µg/ml *T. polium* and their combination. The plates were incubated for 72h and were then stained with AO/EB dye mixture. (100 µg/ml of AO and 100 µg/ml of EB in PBS). The cells were observed under a fluorescent microscope.^30^

### NO measurement 

Griess reaction was used to determine NO using colorimetric detection of nitrite. HUVECs were cultured in 24 well plates and incubated for 24 h. The cells were then treated with 300 µM tranilast, 100 µg/ml *T. polium* and their combination of for 72h. The absorbance was measured using an ELISA reader at 540 and a reference of 630 nm.^31^

### Migration assay 

*In vitro* scratch assay was carried out to examine the effect of *T. polium* and/or tranilast on the migratory ability of HUVECs. Cells were cultured in 4-well plates and allowed to form a confluent monolayer. Wounds were made with a plastic tip (1 mm width) and debris was removed by washing with phosphate buffered saline (PBS). The plates were incubated for 72 h and the artificial scratch was then photographed.by an inverted microscope equipped with a digital camera, The length of the wound was then chosen for photograph. After photography, medium containing *T. polium* and/or tranilast were added. Finally, the images were analyzed using TScratch software, Version 1.0 (MathWorks Inc).^32^

### Real time RT-PCR 

Total RNA was isolated from HUVECs using the GeneMatrix Universal RNA Purification Kit (EURx Ltd. Gdansk Poland ul), according to the manufacturer’s instructions. The isolation was performed in three independent replicates for each experiment. Complementary DNA (cDNA) synthesis was carried out using cDNA synthesis kit (PrimeScriptTM 1st strand cDNA Synthesis Kit, Takara) in 20 µl reaction mixture according to the manufacturer’s instructions. Real-time PCR was performed using SYBR Premix Ex Taq technology (Takara Bio Inc. Japan) on the Applied Biosystems Real-Time PCR One step System in triplicate. Glyceraldehyde 3-phosphate dehydrogenase (*GAPDH*) was used as an internal control. The primers were designed using the Bio Edit and BLAST program. (http://www.ncbi.nlm.nih.gov) After analyzing the data using One Step Software v3.2, the relative expression level of *BAX* and *BCL-*_2_ genes was calculated using two ^-ΔΔCT^ formula. The primers were designed using the program Bio Edit, BLAST searches (http://www.ncbi.nlm.nih.gov) performed to confirm specificity of the selected nucleotide sequences.The sequences of the reverse transcription (RT) primers used were: *BAX* forward: TGTTTGCTGATGGCAACTTC and reverse: GATCAGCTCGGGCACTTTAG*; BCL-2* forward: GGGATGCCTTTGTGGAACTA and reverse: CTCACTTGTGGCCCAGGTAT*; GAPDH* forward: ACTCTGGTAAGTGGATATTGTTC and reverse: GGAAGATGGTGATGGGATTTC.

### Statistical analysis 

One-way analysis of variance ANOVA test was performed to determine the significance of differences among groups using SPSS statistical software (version 16.0 SPSS Inc.). All data were shown as means ± standard error of mean (SEM) and values of p<0.05 were considered significant.

## Results

### Effects of T. polium, tranilast , and their combination on the viability of HUVECs

**Figure 1:**
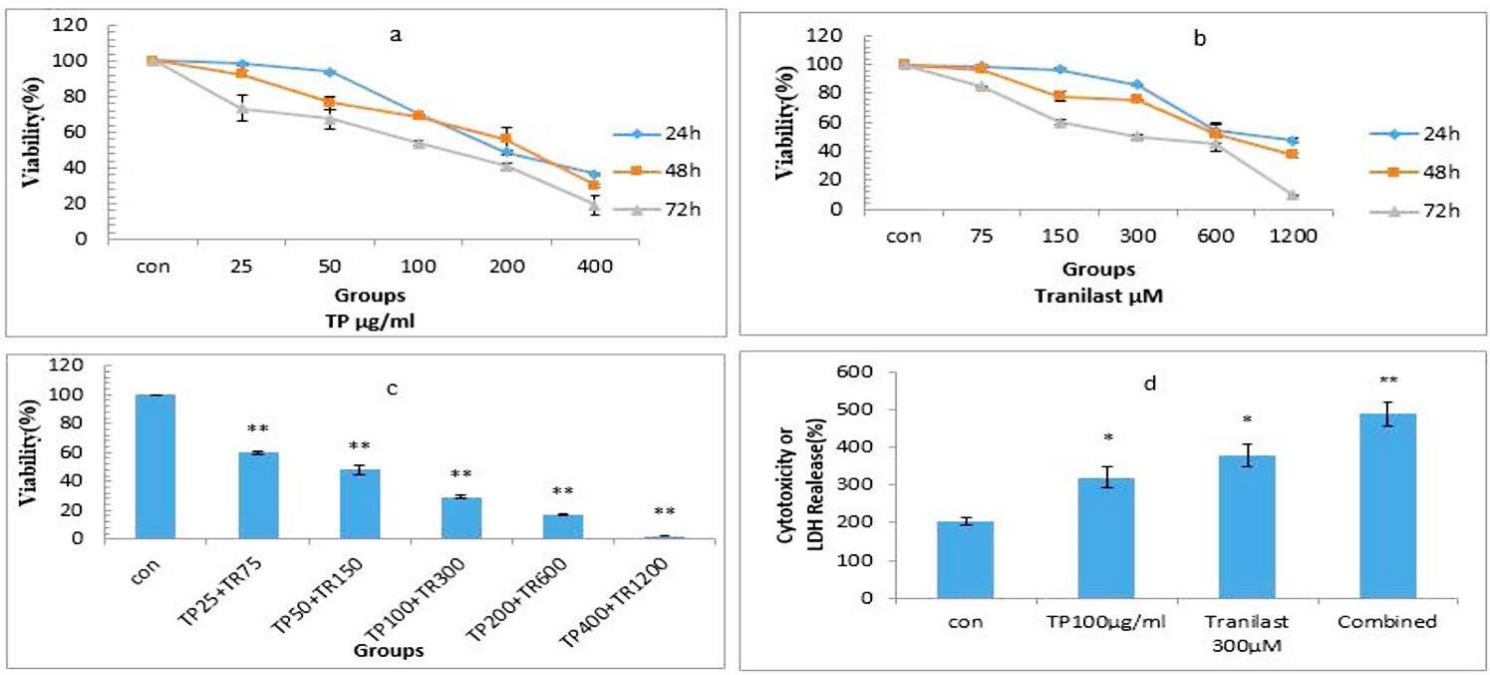
Effects of *T. polium* (a) and tranilast (b) alone and in combination (c) on HUVECs viability and toxicity using MTT and Lactate Dehydrogenase (LDH) assay (d). Cells were treated with *T. polium* and/or tranilast for 24, 48 and 72h and viability was measured by MTT assay. Cells were treated with *TP* and/or Tranilast for 72h and toxicity was measured by LDH assay. Control wells were treated with equivalent amount of medium alone. The values are the mean and SE from triplicated experiments.*TP*: *Teucrium polium* (*P < 0.05; ** P < 0.001 compared with control)

The cell viability of HUVECs was decreased from 98.33± 0.88 to 36.66± 0.88, 92.33± 1.76 to 30.33± 0.88 and 73.33± 7.35 to 19.33± 5.20%, respectively, with increase in doses of *T. polium* (25, 50,100.200,and 400 µg/ml), after 24, 48 and 72 h. In addition, incubation of HUVECs by increasing the concentrations of tranilast ‏)75, 150, 300, 600,and 1200 µM) resulted in a decrease in cell viability from 98.66± 0.88 to 47.33± 2.02, 96.33± 0.88 to 37.33± 2.02 and 84.66± 0.88 to 9.66± 0.88 %, respectively after 24, 48 and 72 h incubation (Figure 1).

IC50s for *T. polium* and tranilast were about 100 µg/ml and 300 µM, respectively, after 72h, Therefore, 100 µg/ml *T. polium* was used in combination treatment with 300 µM tranilast and cell viability was assessed after 72 h. Combined treatment decreased the cell viability much higher than using either of the agents alone (Figure 1). Combination index (CI plot‏ (, and‏ dose reduction index‏) DRI ‏(, were then calculated.The CI values in all combinations were smaller than one, implying a synergistic effect in all combination tests. The DRI values for both *T. polium* and tranilast were greater than one showing a dose reduction for a given therapeutic effect in both of them (Table 1 and 2).

**Table 1:** Combination indexa (CI) values for *T.polium* and Tranilast combination

**T.Polium(µg/ml)**	**Tranilast(µM)**	**CI**	**Interpretation**
25	75	0.71	Synergism
50	150	0.81	Synergism
100	300	0.87	Synergism
200	600	0.92	Synergism
400	1200	0.21	Synergism

**Table 2:** Dose reduction indexa (DRI) values for *T.polium* and Tranilast combination

**Fa**	**Dose T.polium**	**Dose Tranilast**	**DRI T.polium**	**DRI Tranilast**
0.41	68.86	214.5	2.75	2.86
0.55	132.46	341.3	2.64	2.27
0.7	280.2	580.99	2.80	1.93
0.82	608.34	1007.16	3.04	1.67
0.98	9538.88	7102.65	23.84	5.91

### Effects of T. polium, tranilast and their combination on LDH activity of HUVECs

Measurement of LDH activity revealed that *T. polium* and/or tranilast significantly increased LDH release after 72 h incubation. Combination of *T. polium* and tranilast significantly increased medium LDH activity (p<0.001) more than either *T. polium* or tranilast alone p<0.05 (Figure 1).

### Effects of T. polium, tranilast and their combination on HUVECs’ apoptosis 

**Figure 2:**
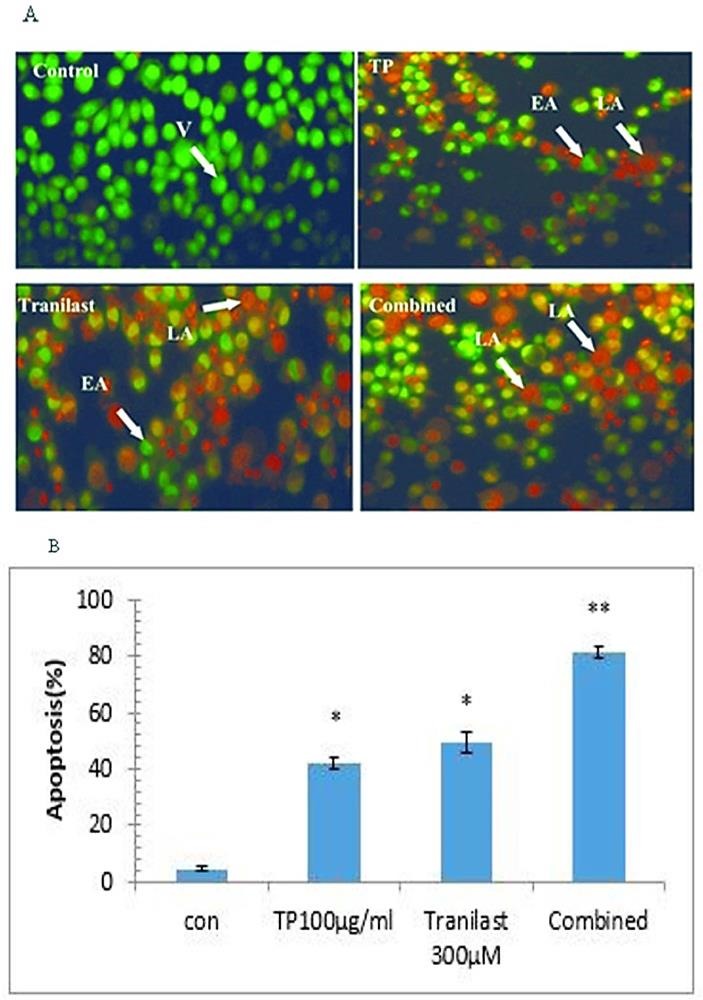
Effects of *T. polium,* tranilast and their combination on apoptosis in HUVECs stained with AO/EB after 72 h. a: Fluorescence Micrographs of cells in control, *T. polium*, tranilast and their combination after 72h. V: viable, EA: early apoptotic, LA: late apoptotic magnification 400x. b: Quantitation of apoptotic HUVEC cells. The values are expressed as mean±S.E. ^*^p<0.05 and ^**^p<0.001. *TP:Teucrium polium*. Con: Control

Apoptosis induction was studied using AO/EB staining in HUVECs treated with *T. polium*, tranilast and their combination after 72 h incubation. As shown in Figure 2A, morphological changes of apoptotic cells include chromatin condensation and cell shrinkage as compared to the control cells. The level of apoptotic HUVECs were 42± 2.30 in 100 µg/ml *T. polium* and 49.3± 3.71% in 300µM tranilast (p<0.001). The data showed that HUVECs were more sensitive to combined treatment (81.3± 1.76%) as compared to each drug alone (Figure 2B). By referring to Figure 2A, it was discovered that both early and late apoptotic cells were detected in the presence of 100 μg/ml *TP* and 300‏-µM tranilast. In the combined treatment, nearly all of the cells were at late apoptotic phase.

### Effects of T. polium, tranilast and their combination on NO production

**Figure 3:**
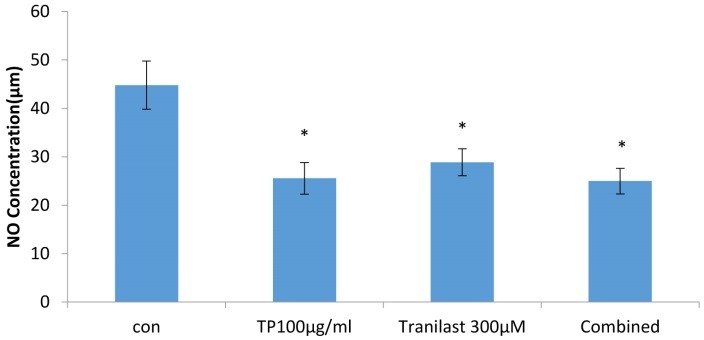
The effects of *T. polium* and/or tranilast on nitric oxide (NO) production in HUVECs. Cells were treated with *T. polium*, tranilast and their combination for 72 h and nitric oxide concentration was measured using Griess assay. Control wells were treated with equivalent amount of medium. The values are mean±SD from triplicated experiments. (*P<0.05 compared to the control). *TP: Teucrium polium*. Con: Control

The findings of Griess method showed that *T. polium* (P= 0.002) and tranilast (P= 0.01) significantly decreased the NO secretion of HUVECs cells after 72 h compared with the control. Furthermore, *T. polium*/Tranilast combination significantly reduced NO content, the same content for each drug alone (P=0.002) (Figure 3).

### Effects of T. polium, tranilast and their combination on migration potential of HUVECs’ 

**Figure 4:**
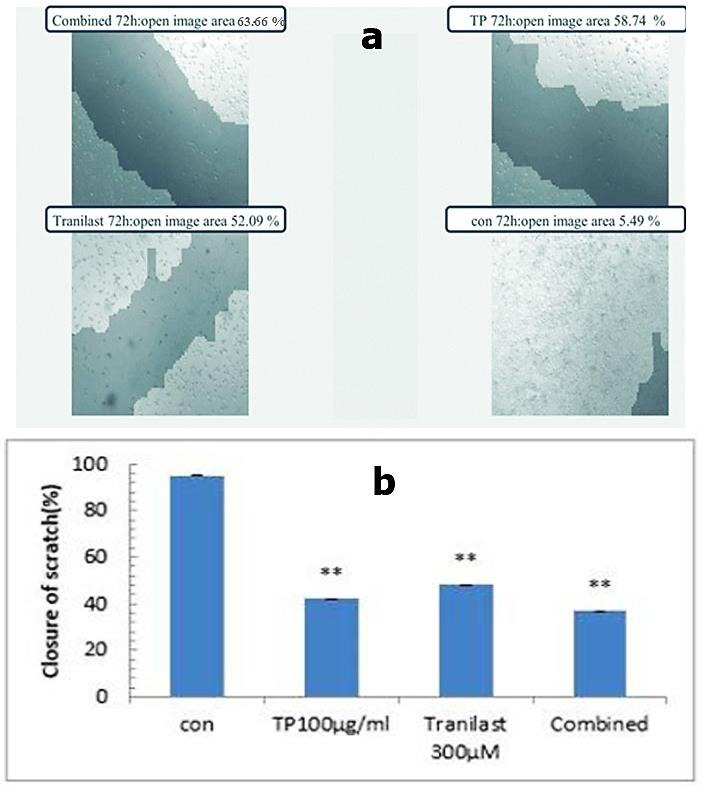
The effects of *T. polium*, tranilast and their combination of them on migration in HUVECs’ line. Confluent cells were scratched and then treated with *T. polium*, tranilast and their combination of them for 72 h. Closure of the scratches was photographed (a) and calculated (b) as the percentage of migration. ( ** p<0.001 vs. control). *TP: Teucrium polium*. Con: Control

To evaluate the effects of *TP* and tranilast as a single and/or combined treatment on cell migration, wound healing (scratch) assay in HUVECs was performed. After 72 h treatment, cells in the control group efficiently spread into the wound area to such an extent that the wound boundary was almost indistinguishable, whereas a low number of cells in *TP* or tranilast treated groups crossed the artificial wound boundary. The results clearly showed that *T. polium*(P= 0.00) and tranilast (P=0.00) alone and in combination (P=0.00) significantly decreased the migration of HUVECs (Figure 4).

### Effects of T. polium , tranilast and their combination on the expression of BAX and BCL-2

**Figure 5:**
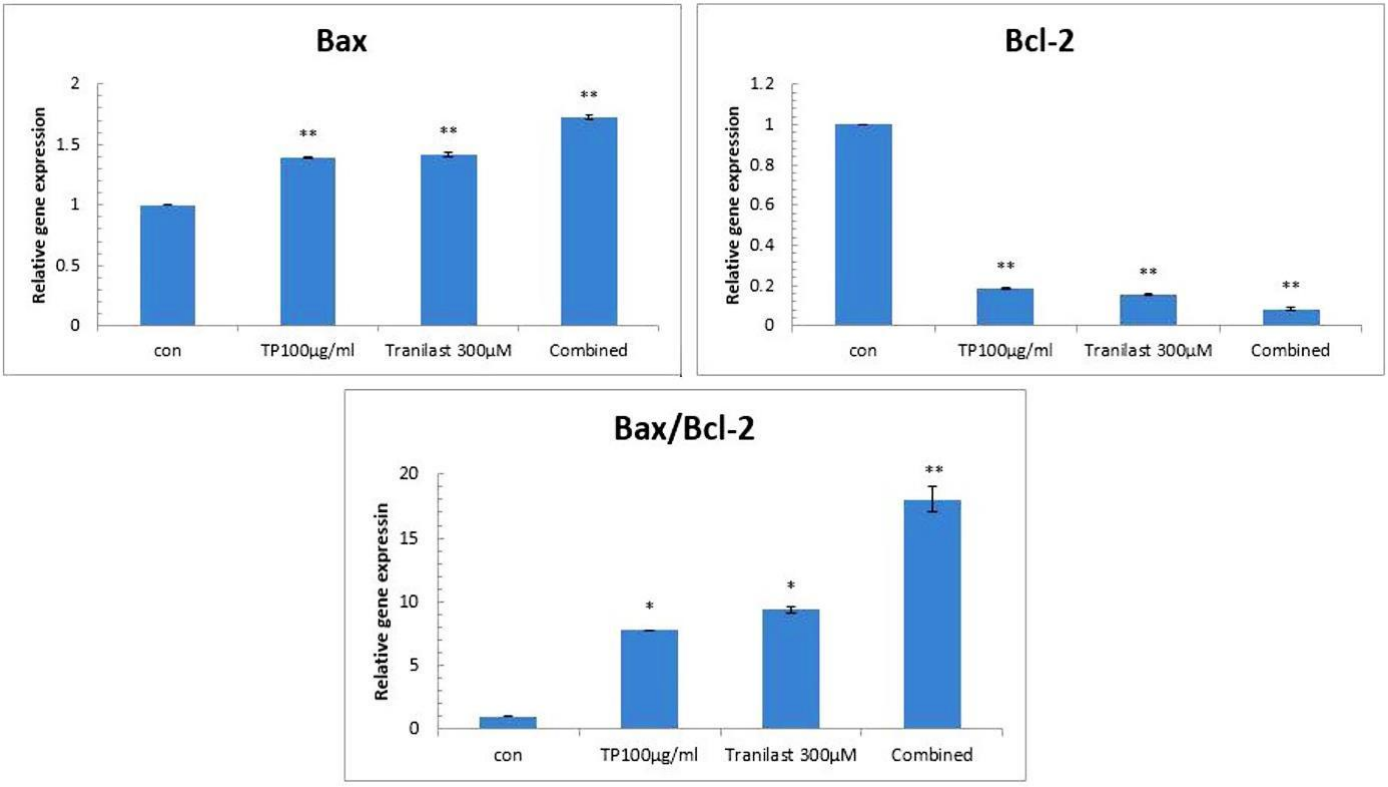
Effects of *TP*, Tranilast and their combination on mRNA expression of *Bax* and* Bcl-2* genes of HUVECs using real-time RT-PCR after 72h. Quantitative RT-PCR was done in HUVECs treated by *TP*, tranilast and combination of them. A significant increase in *BAXx/BCL-2* ratio was observed with combined treatment of TP and Tranilast, respectively, compared to the control. The values are expressed as mean±S.E. ^*^p<0.05 and ^**^p<0.001. *TP:Teucrium polium*. Con: Control

To investigate the mode of action of *T. polium*, tranilast and their combination on cell death, *BAX* and *BCL-2* mRNA expression was evaluated using quantitative real time PCR.* T. polium* , tranilast and their combination increased the expression of *BAX,* a pro-apoptotic molecule, and decreased the expression of *BCL-2*, an anti-apoptotic molecule in HUVECs after 72 h. (p<0.001) when compared with the control. As shown in Figure 5, *BAX* / *BCL-2* mRNA ratios were7.7 in *T. polium TP* (P=0.003), 9.3 in tranilast (P= 0.001) and 18 in combined group (P=0.000). Thus, up-regulation of *BAX* and down-regulation of *BCL-2* mRNAs observed in this study may be one of the fundamental mechanisms through which *T. polium* and/or tranilast induces apoptosis in HUVECs.

## Discussion

In this study, the synergic effect of *T. polium*, a medicinal plant, and tranilast on HUVECs was studied for the first time. *T. polium* and tranilast alone, and in combination reduced the viability of HUVECs in a dose-and time-dependent manner. Combined treatment significantly increased cytotoxicity with a combination index value between 0.71 and 0.21, showing synergistic effects of* T. polium* and tranilast that were in line with LDH assay.

Petroleum ether fraction of *T. polium* has been reported as the most active fraction of *T. polium* on U87 cells.^22^ The most anti-proliferative properties of *T. polium* are believed to be related to terpenoids and flavonoids. These compounds in *T. polium* extract are able to influence a variety of cell functions by inhibiting cell proliferation and migration.^19^

Tranilast inhibits the proliferation of human coronary smooth muscle cells (SMCs) in a dose dependent manner. Cell-cycle analysis using flow cytometry showed that tranilast arrests the SMC cell cycle at G1 in growing-phase cells and inhibited the cell-cycle progression from G0:G1 to S phase in G0-arrested cells.^33^ To understand the possible mechanisms involved in the antiangiogenic activity of tranilast and *T. polium* combination, the rate of apoptosis in HUVECs was calculated. According to the AO/EB staining results, tranilast and *T. polium* combination significantly induced apoptosis in HUVECs, when compared with the untreated cells and single drug treatments.

The results were consistent with previous study that *T. polium*/vincristine and *T. polium*/vinblastine combinations treatment has induced apoptosis and provoked tumor cell killing in several human cancer cell lines such as MCF7, A431, SW480, Skmel3 and EJ. In agreement with the results, combination of *T. polium* and doxorubicin resulted in a massive apoptosis compared with the effect of individual drugs. Moreover, combinations with *T. polium* extract reduced the cytotoxic effects of known anticancer drugs.^18^ Some studies have also shown the ability of tranilast to induce apoptosis in MDA-MB-231 and MCF-7 cell lines. It is the fact that AO/EB staining is a convenient way to diagnose the appearance of dense chromatin, the nuclear fragmentation and the apoptotic bodies in tranilast treated cells.^34^

To elucidate the underlying mechanism involved in the apoptotic properties of tranilast, *T. polium*, and their combination, the expression of apoptosis-related genes have beed evaluated. *BAX* is a proapoptotic *BCL-2* family member that plays an important role in induction of mitochondrial dependent apoptosis. On the other hand, *BCL-2* is an antiapoptotic member that can counteract *BAX* function in initiation of cell death. Modulations of these factors to inhibit or stimulate apoptosis determine the fate of the cells to die or survive.^35^

Some alterations were determined in the expression of *BAX* and *BCL-2* after different treatments. Combination of tranilast and *T. polium* resulted in upregulation of *BAX* and downregulation of *BCL-2*, and an increased ratio* of BAX/BCL2*. The findings of the present study are supported the experiments by Haïdara et al. who reported an increase in *BAX/BCL2* ratio following treatment of MCF-7 and MDA-MB-231 by combination of tranilast and tamoxifen. Flavonoid compounds in *T. polium* also have antiproliferative property that can induce apoptosis via *P53* and other regulators of cell apoptosis.^36^ Therefore, cell death induced by *T. polium* extract could be related to *P53* activation and other regulators of cell death and apoptosis.

In mammals including humans, NO is an important cell-signaling molecule involved in many physiological and pathological processes. NO is a mediator of angiogenesis as well.^37^ *T. polium* and/ or tranilast in our experiments significantly reduced NO production in HUVECs. Combination of 100 µg/ml *T. polium* and 300-µM tranilast had more profound effect on NO production in HUVECs. It has been reported that tranilast is able to inhibit NO release from N9 microglial cells via suppression of iNOS mRNA expression and iNOS protein accumulation.^38^

The present experiments clearly showed that *in vitro* combination therapy of *T. polium* and tranilast could significantly increase anti-angiogenic properties of these agents in an *in vitro* model of angiogenesis (HUVECs) cells. However, *in vivo* studies on solid tumors in a suitable animal model could highlight the probable potential of *T. polium* /tranilast combination therapy as an antiangiogenic therapy procedure in solid tumors.

## Conclusion

These results showed that *T. polium* (*TP*) plus tranilast could be a promising combination therapy for future clinical trials in cancer patients through angiogenesis inhibition. Combination of *T.polium* and tranilast can induce anti-angiogenic effects on HUVECs, which in turn lead to reduced cell viability, increased apoptosis and decreased migration capacity of these cells. Further *in vivo* studies and future clinical trials are proposed to evaluate the efficacy of *T. polium* and tranilast combination therapy for angiogenesis inhibition as a new therapeutic option.

## Acknowledgments

This study was part of Ph.D. thesis. The authors appreciate the financial support of this investigation by Kerman and Kermanshah University of Medical Sciences. The authors would like to thank Radan native english edit for editing the manuscript.

## Ethical Issues

This experimental *in vitro* study was carried out using HUVECs. This study was approved by ethics committee at Kerman University of Medical Science. Ethical code: IR. KMU.REC.1395.152.

## Conflict of Interest

The authors declare that they have no competing interest.
